# Screening and identification of muscle pericyte selective markers

**DOI:** 10.1038/s41598-025-14225-3

**Published:** 2025-08-07

**Authors:** Jingsong Ruan, Minkyung Kang, Rong Wang, Wanling Xuan, Feng Cheng, Yao Yao

**Affiliations:** 1 Department of Molecular Pharmacology and Physiology, Morsani College of Medicine, University of South Florida, 12901 Bruce B. Downs Blvd., MDC 8, Tampa, FL 33612 USA; 2 Department of Pharmaceutical Sciences, Taneja College of Pharmacy, University of South Florida, Tampa, FL USA; 3 Present Address: Department of Neurosurgery, Stanford University, Stanford, California, FL USA

**Keywords:** Pericytes, Smooth muscle cells, Genetic tool, PDGFRβ, SM22α, Cell biology, Computational biology and bioinformatics, Molecular biology

## Abstract

Pericytes, which share markers with smooth muscle cells (SMCs), are heterogenous cells. Pericytes in the brain and skeletal muscle have different embryonic origins, representing distinct subpopulations. One challenge in the field is that there are no subpopulation-specific pericyte markers. Here, we compared the transcriptomes of muscle pericytes and SMCs, and identified 741 muscle pericyte-enriched genes and 564 muscle SMC-enriched genes. Gene ontology analysis uncovered distinct biological processes and molecular functions in muscle pericytes and SMCs. Interestingly, the Venn diagram revealed only one gene shared by brain and muscle pericytes, suggesting that they are indeed distinct subpopulations with different transcriptional profiles. We further validated that GSN co-localized with PDGFRβ^+^SMA^−^ cells in small and large blood vessels but not PDGFRβ^+^SMA^+^ cells, indicating that GSN predominantly marks pericytes and fibroblasts rather than SMCs in skeletal muscle. Negligible levels of GSN were detected in the brain. These findings indicate that GSN may serve as a selective marker for muscle pericytes.

## Introduction

Pericytes and smooth muscle cells (SMCs), together known as mural cells, are perivascular cells, which cover different segments of the vascular tree^1^. Specifically, SMCs reside in large blood vessels, while pericytes predominantly cover small blood vessels^1^. Biochemically, these cells are very similar and share many cellular markers. For example, the most widely used pericyte markers, such as PDGFRβ and CD13, are also expressed in SMCs^1,2^. Although several SMC-specific markers, including SM22α^3,4^ and Myh11^5^, are available, there are few pericyte-specific markers currently^1^. In addition, pericyte heterogeneity has been reported^1,6,7^. First, pericyte number varies significantly across different tissues. It has been shown that pericyte density is much higher in the brain compared to skeletal muscle^1,8,9^which is consistent with their crucial function in blood-brain barrier (BBB) integrity^1,10,11^. Next, the embryonic origins of pericytes in different tissues vary. For example, brain pericytes originate from ectoderm-derived neural crest^1,12–15^while skeletal muscle pericytes have a mesoderm origin^1,16,17^. This inter-tissue heterogeneity likely represents different subpopulations, which may exert tissue-specific functions. Unfortunately, the biochemical properties and functions of these subtypes of pericytes remain largely unknown, mainly due to the lack of subpopulation-specific pericyte markers.

To fill this critical gap of knowledge, researchers have started to investigate pericyte biology and function in a tissue-specific manner. In the brain, pericytes exert a variety of important functions, including BBB maintenance^10,11,18^ and blood-flow regulation^19–22^. In skeletal muscle, pericytes may function as progenitor cells for myoblasts and/or other cells^16,23,24^. Under pathological conditions, pericytes may acquire stem/progenitor properties and contribute to tissue regeneration or fibrosis depending on the conditions^23,25–27^. Recently, several brain pericyte-selective markers, including *Kcnj8*, *Abcc9*, and *Atp13a5*, have been identified^28,29^. By comparing the transcriptomes of brain pericytes and SMCs, we obtained 40 brain pericyte-enriched genes with many reported in previous studies and identified new brain pericyte-selective markers in a recent study^30^. It remains unclear whether these brain pericyte-selective markers can label pericytes in other tissues. Unlike brain pericytes, no muscle pericyte-selective markers have been reported, although several non-specific markers (PDGFRβ and NG2) have been used to identify muscle pericytes^16,31^.

In this study, we screened and identified muscle pericyte-selective markers by RNAseq analysis using skeletal muscles from mice expressing tdTomato in SMCs. Since brains from the same mice were used to determine brain pericyte-selective markers, which substantially diminished individual differences and system variations, we further cross-compared brain and muscle pericyte-selective markers.

## Results

### Isolation of pericytes and SMCs from mouse skeletal muscle

**Fig. 1:**
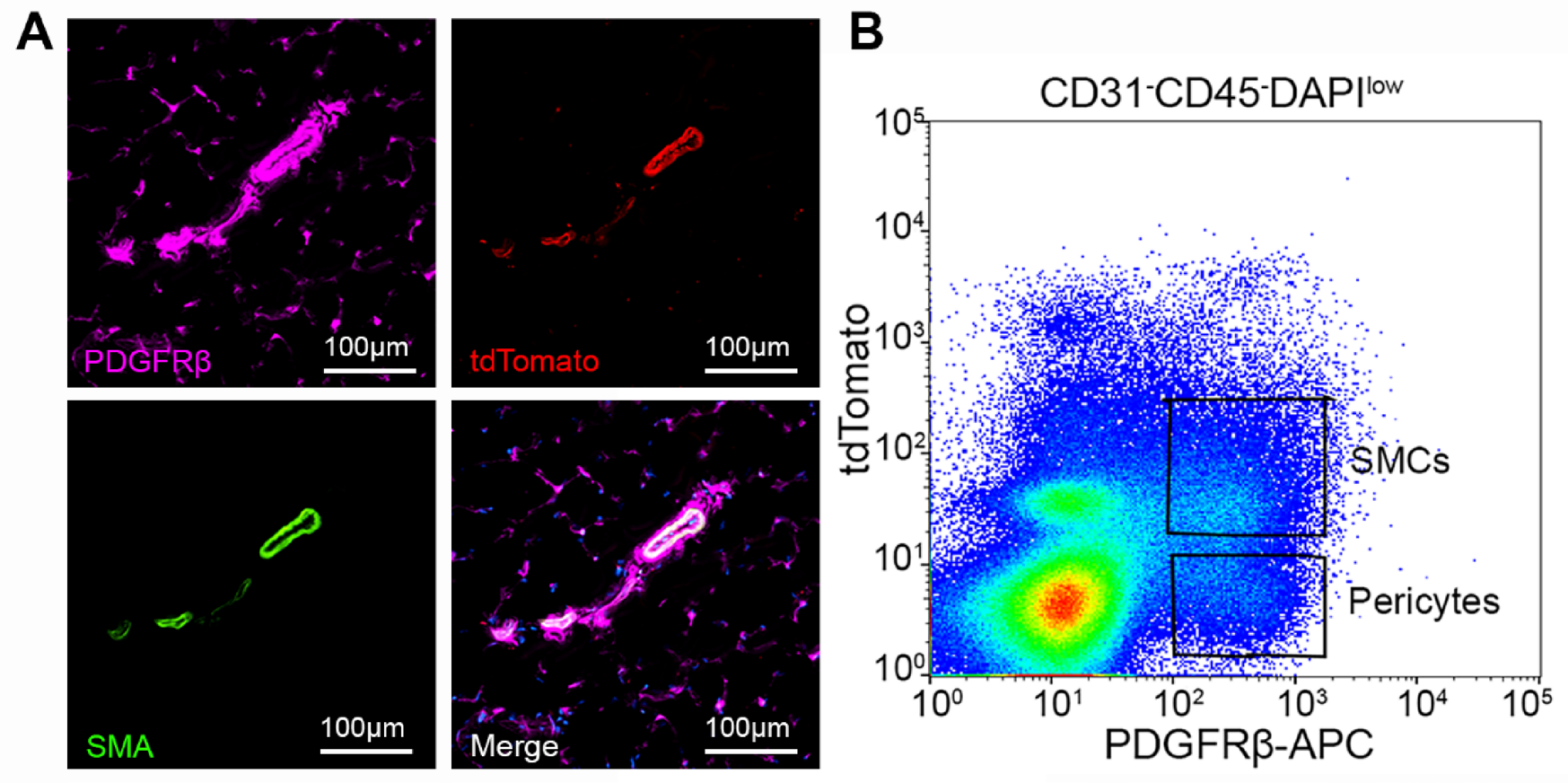
Validation and isolation of muscle pericytes and SMCs from Ai14^+/−^;SM22α-Cre^+^ mice. (**A**) tdTomato and SMA co-localized with PDGFRβ in large but not small blood vessels in mouse TA muscle. (**B**) Representative flow cytometry plot showing pericytes (PDGFRβ^+^tdTomato^−^) and SMCs (PDGFRβ^+^tdTomato^+^).

To enable live sorting of SMCs, we generated Ai14^+/−^;SM22α-Cre^+^ mice, in which SM22α^+^ SMCs are permanently labeled with tdTomato. Immunohistochemistry (IHC) revealed a perfect co-localization of tdTomato with smooth muscle actin-α (SMA) in tibialis anterior (TA) muscle (Fig. 1A). Additionally, tdTomato and SMA signals partially co-localized with PDGFRβ (mural cell marker) with tdTomato and SMA expression in large but not small blood vessels (Fig. 1A). These results demonstrate the feasibility to distinguish SMCs (PDGFRβ^+^tdTomato^+^ cells) and pericytes (PDGFRβ^+^tdTomato^−^ cells) with PDGFRβ and tdTomato.

Next, we isolated pericytes and SMCs from skeletal muscles of Ai14^+/−^;SM22α-Cre^+^ mice by flow cytometry. After excluding CD31^+^ endothelial cells, CD45^+^ hematopoietic cells, and DAPI^high^ dead cells, pericytes and SMCs were gated based on PDGFRβ and tdTomato expression. The workflow and gating strategy are shown in Figure S1. We observed two populations: PDGFRβ^+^tdTomato^−^ and PDGFRβ^+^tdTomato^+^ cells representing pericytes and SMCs, respectively (Fig. 1B). To improve visualization and better illustrate the populations, the dot plot and contour plot are also provided (Fig. S1). These results demonstrate that we can successfully isolate pericytes and SMCs from skeletal muscles using this approach.

### RNAseq analysis of muscle pericytes and SMCs

**Fig. 2:**
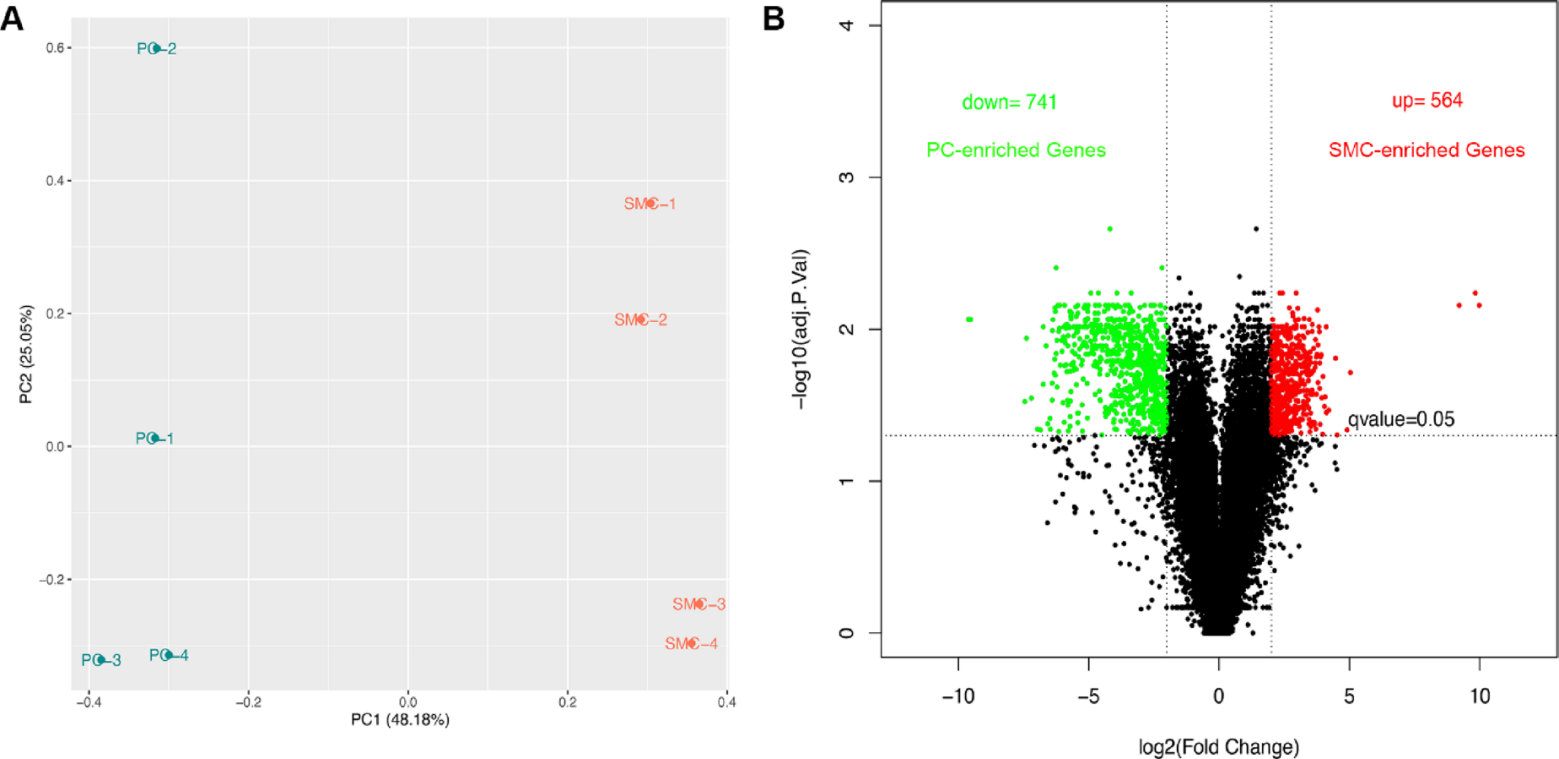
RNAseq analysis of freshly isolated muscle pericytes and SMCs. (**A**) The PCA plot showed that muscle pericytes and SMCs were clearly separated, although some variations were observed among biological replicates. (**B**) The volcano plot revealed 741 muscle pericyte-enriched genes and 564 muscle SMC-enriched genes. PCA, principal component analysis.

To screen for muscle pericyte-specific markers, we performed bulk RNAseq analysis using pericytes and SMCs freshly isolated from skeletal muscles of Ai14^+/−^;SM22α-Cre^+^ mice. The principal component analysis (PCA) showed that pericyte and SMC samples were clearly separated from each other, although some variations existed among biological replicates (Fig. 2A). These variations may be caused by individual differences and batch effects, since multiple sorts were combined to obtain sufficient cells for one sample due to the relatively low density of pericytes in skeletal muscle^1^. This finding indicates that muscle pericytes and SMCs are transcriptionally different.

**Fig. 3:**
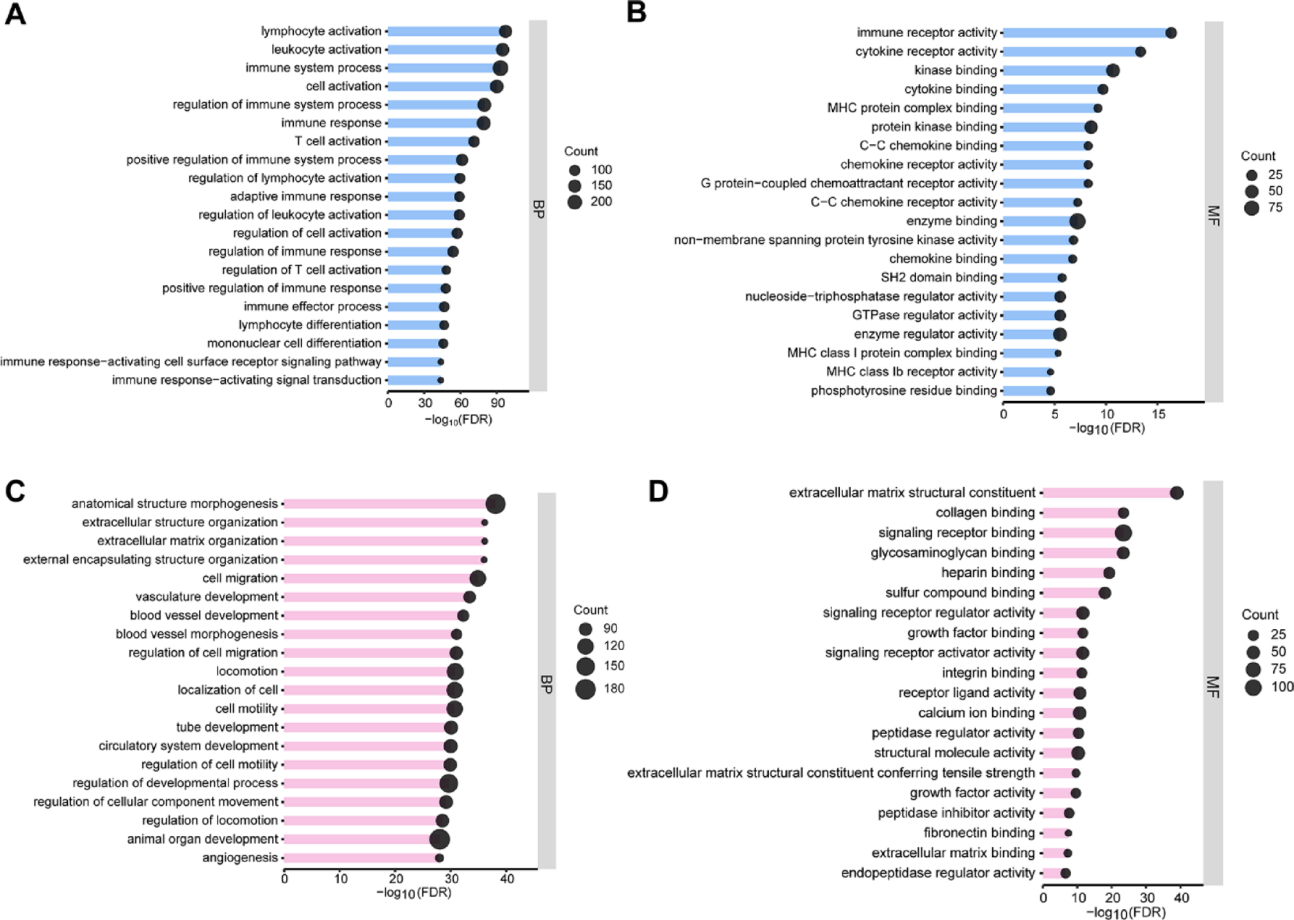
Muscle pericyte-enriched and SMC-enriched genes are involved in distinct biological processes and molecular functions. (**A** and **B**) Gene ontology analysis demonstrating major biological processes (**A**) and molecular functions (**B**) in muscle pericytes. (**C** and **D**) Gene ontology analysis demonstrating major biological processes (**C**) and molecular functions (**D**) in muscle SMCs.

Next, we compared the transcriptional profiles of muscle pericytes and SMCs, and identified 1305 differentially expressed genes (DEGs), defined as those with Log2FC more than 2 and q value less than 0.05 (Table S1). Among these DEGs, 741 were up-regulated in muscle pericytes and 564 were up-regulated in muscle SMCs (Fig. 2B). To identify the biological processes and molecular functions enriched in these cells, we performed gene ontology analysis using these DEGs. Muscle pericyte-enriched biological processes include lymphocyte activation, leukocyte activation, immune system process, cell activation, regulation of immune system process, immune response, and T cell activation (Fig. 3A). Consistent with this finding, immune-related molecular functions, including immune receptor activity, cytokine/chemokine receptor activity, cytokine/chemokine binding, MHC protein complex binding, G protein-coupled chemoattractant receptor activity, and kinase/enzyme binding, were enriched in muscle pericytes (Fig. 3B). These results highlight a relative enrichment of immune response/activation pathways in muscle pericytes compared to muscle SMCs.

Compared to muscle pericytes, very different biological processes and molecular functions were enriched in muscle SMCs. Specifically, biological process analysis revealed that anatomical structure morphogenesis, blood vessel development/morphogenesis, animal organ development, extracellular structure/matrix organization, and cell migration/motility were enriched in muscle SMCs (Fig. 3C). Molecular function analysis showed that extracellular matrix structural constituent, collagen binding, signaling receptor binding, glycosaminoglycan binding, heparin binding, sulfur compound binding, growth factor binding, and integrin binding were enriched in muscle SMCs (Fig. 3D). These results highlight an essential role of muscle SMCs in tissue development/morphogenesis and extracellular matrix organization.

### Identification of muscle pericyte-selective markers

**Fig. 4:**
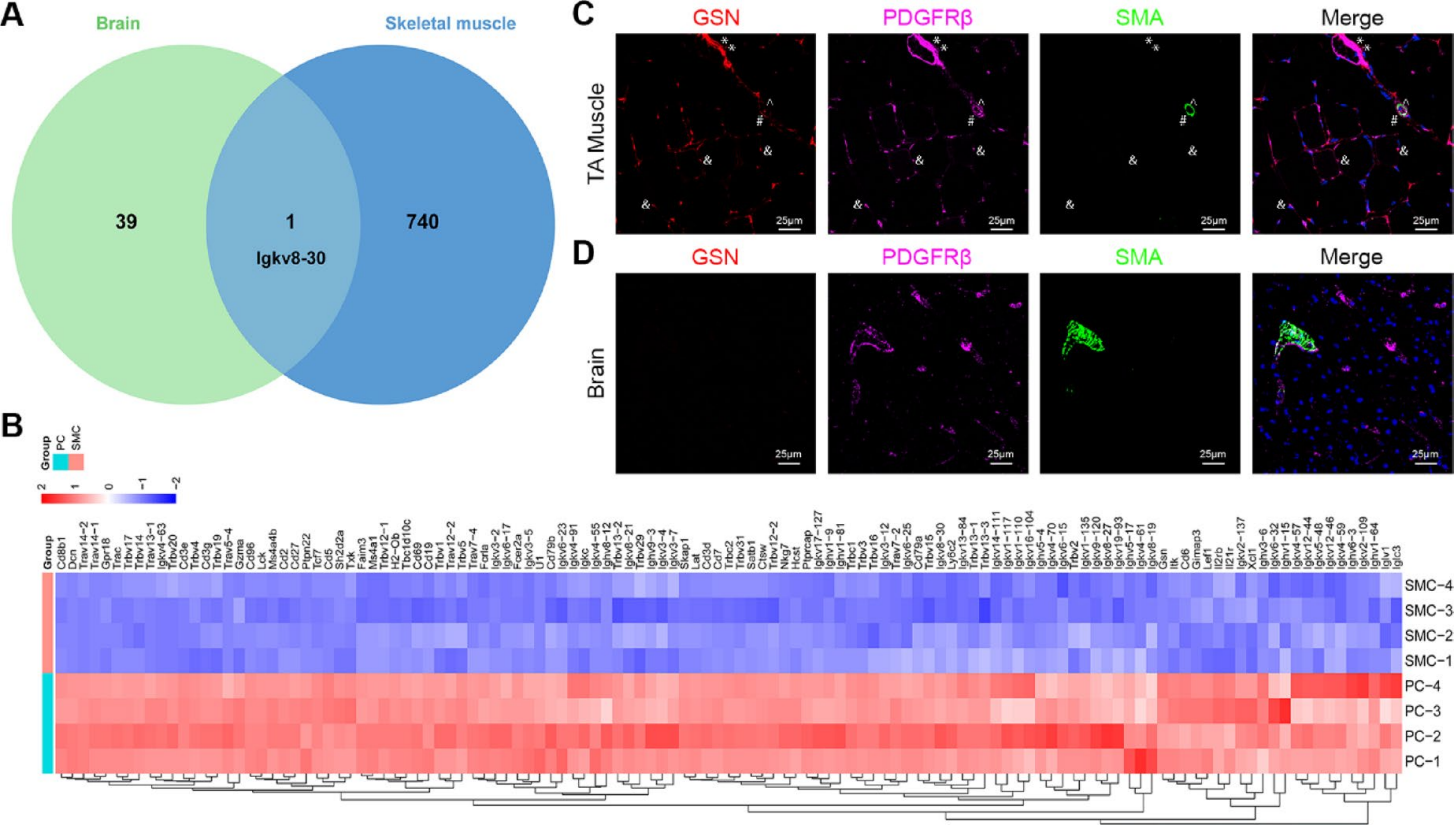
Validation of muscle pericyte-enriched genes in vivo. (**A**) Venn diagram showing only one overlapping gene between brain and muscle pericyte-enriched genes. (**B**) Heatmap showing top 121 genes (Log2FC more than 5 and q value less than 0.05) enriched in muscle pericytes compared to muscle SMCs. (**C**) GSN was detected in PDGFRβ^+^SMA^−^ pericytes (&) in capillaries and PDGFRβ^+^SMA^−^ fibroblasts in large vessels (* and #), but not PDGFRβ^+^SMA^+^ SMCs (^) in arteries/arterioles in mouse TA muscle. (**D**) GSN was not detected in mouse brain.

To determine the transcriptional differences of pericytes in different tissues, we compared muscle pericyte-enriched genes with brain pericyte-enriched genes reported in a previous study from our laboratory^30^. This study was chosen for two reasons: (1) brains used in the previous study and skeletal muscles used in the current study were from the same Ai14^+/−^;SM22α-Cre^+^ mice, and (2) brain and muscle tissues were processed and analyzed simultaneously using the same pipeline, which significantly reduced variations and batch effects. Compared to the 40 brain pericyte-enriched genes reported previously, 741 muscle pericyte-enriched genes were identified (Fig. 2B). The significantly higher number indicates tissue-specific signature of pericytes. Venn diagram revealed only one shared gene (Igkv8-30) between brain and muscle pericyte-enriched genes with 39 and 740 genes unique to brain and muscle pericytes, respectively (Fig. 4A), again suggesting that brain and muscle pericytes are different subpopulations.

Next, we applied more stringent criteria (Log2FC more than 5 and q value less than 0.05) and identified 121 genes highly enriched in muscle pericytes compared to muscle SMCs (Fig. 4B). Among the top 15 genes enriched in muscle pericytes, 12 were immunoglobulin heavy/kappa chain variables and 1 was Trbv17 (T cell receptor beta variable 17), which are highly expressed in the immune system. Among the rest two genes, we chose gelsolin (GSN) to validate since decorin (DCN) is expressed in fibroblasts^32^. GSN is an actin-binding protein, which actively regulates actin filament assembly and disassembly^33^. In TA muscle, GSN co-localized with PDGFRβ^+^SMA^−^ pericytes in capillaries (& in Fig. 4C), but not PDGFRβ^+^SMA^+^ SMCs in arteries/arterioles (^ in Fig. 4C), indicating that GSN selectively labels muscle pericytes rather than SMCs. Interestingly, GSN was also observed in PDGFRβ^+^SMA^−^ cells in the abluminal side of some large veins (* in Fig. 4C) and outside of SMCs in arteries/arterioles (# in Fig. 4C), suggesting that GSN may also mark perivascular fibroblasts in skeletal muscle. In sharp contrast, IHC revealed negligible levels of GSN in the brain (Fig. 4D). This finding is consistent with our RNAseq data showing that GSN is enriched in muscle rather than brain pericytes.

**Fig. 5:**
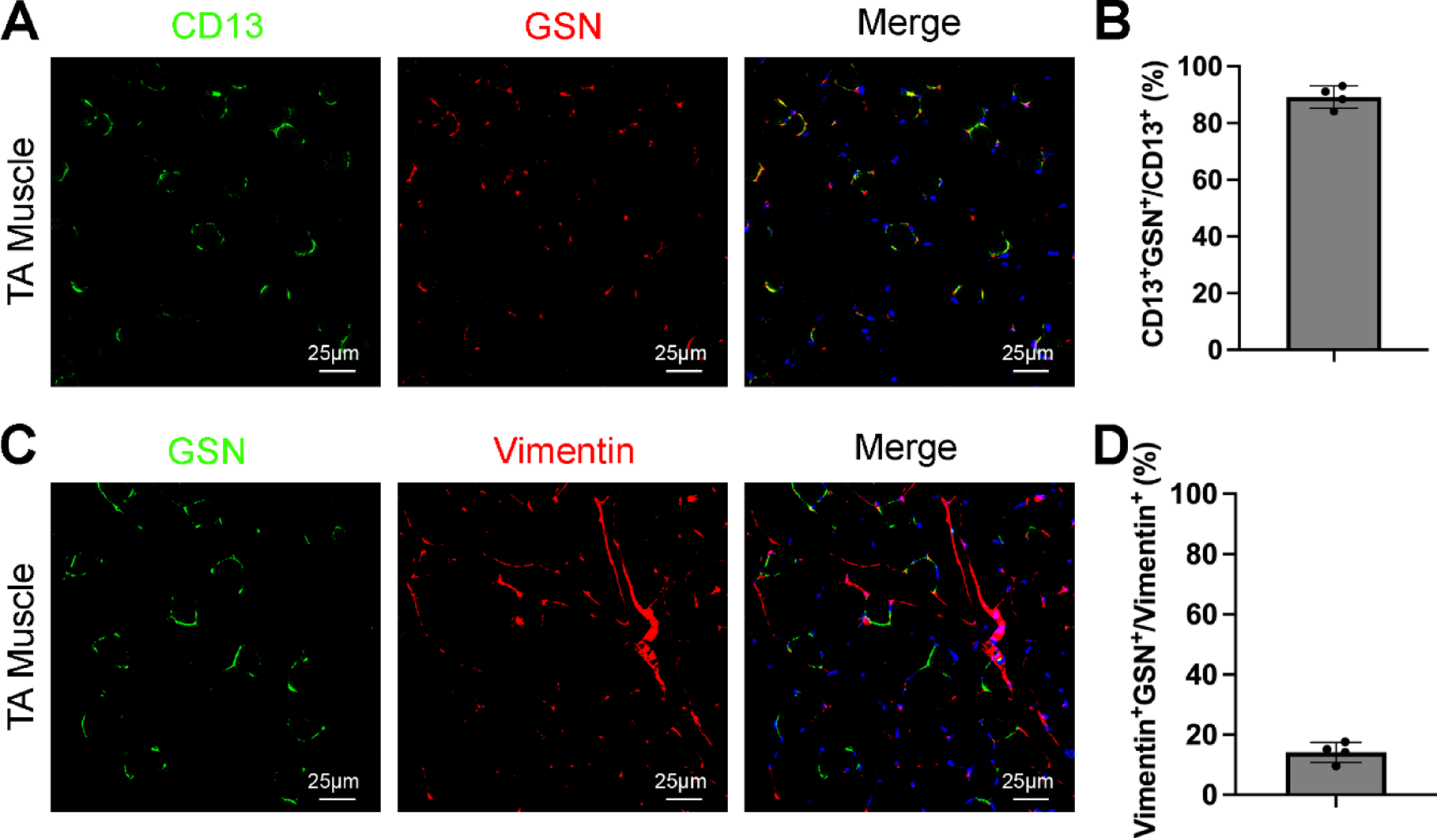
GSN expression in muscle pericytes and fibroblasts. (**A**) Immunohistochemical analysis of GSN and CD13 (pericyte marker) expression in mouse TA muscle. (**B**) Quantification revealed GSN expression in approximately 90% of CD13^+^ pericytes in TA muscle. *n* = 4 mice. Data were shown as mean ± SD. (**C**) Immunohistochemical analysis of GSN and vimentin (fibroblast marker) expression in mouse TA muscle. (**D**) Quantification revealed GSN expression in approximately 14% of vimentin^+^ fibroblasts in TA muscle. *n* = 4 mice. Data were shown as mean ± SD.

To determine if GSN labels all pericytes or a subpopulation of pericytes in skeletal muscle, we co-stained GSN with CD13 (pericyte marker) and quantified the percentage of GSN^+^ pericytes (Fig. 5A). In the TA muscle, approximately 90% of CD13^+^ cells were positive for GSN (Fig. 5B). Given that CD13 also labels SMCs in addition to pericytes^1,2^and that GSN is not expressed in muscle SMCs (Fig. 4C), the actual percentage of muscle pericytes expressing GSN is likely higher than 90%. These results suggest that GSN marks most pericytes in skeletal muscle. Similarly, we also co-stained GSN with vimentin (fibroblast marker) and quantified the percentage of GSN^+^ fibroblasts (Fig. 5C). In sharp contrast, only 14% vimentin^+^ cells expressed GSN (Fig. 5D), indicating that GSN labels a small subpopulation of fibroblasts in skeletal muscle. Together, these results suggest that GSN selectively marks most muscle pericytes and some muscle fibroblasts, but not muscle SMCs or brain pericytes.

## Discussion

Pericytes are perivascular cells that exert a variety of important functions. In the brain, where a stable homeostasis needs to be maintained, pericytes are involved in the regulation of BBB integrity^10,11,18^. In skeletal muscle, which undergoes regeneration after injury, pericytes participate in muscle repair/fibrosis and immunomodulation^1,23,24,34–36^. The tissue-dependent functions of pericytes suggest that pericytes in distinct tissues may have unique transcriptional profiles and markers. Interestingly, various subpopulations of pericytes have been reported even in the same tissues^37–42^. Identifying tissue-specific and/or subpopulation-specific markers for pericytes will substantially enrich our knowledge on pericyte biology/functions.

In this study, Igkv8-30 is identified as the only gene enriched in both muscle and brain pericytes, suggesting that Igkv8-30 may be a universal pericyte marker. It remains unclear if Igkv8-30 is expressed in pericytes in other tissues. It should also be noted that, as an immunoglobulin kappa chain variable, Igkv8-30 is also expressed in immune cells. In addition, we show that GSN labels most muscle pericytes and a small subpopulation of muscle fibroblasts, but not muscle SMCs or brain pericytes. This marker is useful in distinguishing pericytes and SMCs, which share many markers in skeletal muscle^1,2^. It also allows differentiation of brain and muscle pericytes, which are different subpopulations with distinct embryonic origins^1,12–17^. Such subpopulation-specific pericyte markers enable isolation of pericyte subpopulations at high purity and subsequent characterization of their functions.

To the best of our knowledge, this is the first study that cross-compare brain and muscle pericyte-selective markers under the same experimental conditions. Specifically, brain and skeletal muscle samples from the same mice and the same experimental pipeline (flow cytometry followed by RNAseq analysis) were used in these experiments^30^. The same conditions minimize variations in experimental systems and ensure robust data. Interestingly, substantially more pericyte-enriched (741 vs. 40) and SMC-enriched (564 vs. 158) genes were identified in skeletal muscle compared to the brain. This difference suggests that pericytes and SMCs share more features in the brain and may exert more diverse functions in skeletal muscle, reflecting tissue-specific signatures of these cells.

This study has several limitations. First, previous studies have identified two subtypes of pericytes in skeletal muscle: type I (PDGFRβ^+^/NG2^+^/CD146^+^ and Nestin^−^) and type II (PDGFRβ^+^/NG2^+^/CD146^+^ and Nestin^+^)^34,37,38,41,42^. Our IHC revealed GSN staining in most PDGFRβ^+^ pericytes in skeletal muscle, suggesting that GSN may not be able to discern type I and type II pericytes in skeletal muscle. Given the different fates and functions of these cells in muscle regeneration/repair^34,37,39–41^, future research should focus on finding single markers to identify these subtypes of pericytes. Next, although GSN predominantly marks muscle rather than brain pericytes, it remains unclear if it labels pericytes in other tissues, such as liver and kidney. It might be interesting to determine if GSN exclusively labels muscle pericytes. In addition, other than pericytes, GSN also labels PDGFRβ^+^SMA^−^ cells in some large blood vessels (perivascular fibroblasts) in skeletal muscle. Given the overlapping functions between pericytes and fibroblasts in skeletal muscle^1,23,24,34,36,43–45^care should be taken when interpreting results from such studies. Third, due to different pericyte density in tissues and debris levels in samples, muscle and brain pericyte-enriched gene lists are compared directly without performing data integration. The lack of joint normalization and batch correction may introduce statistical biases. Last, this study uses PDGFRβ and SM22α (tdTomato) expression to define pericytes (PDGFRβ^+^tdTomato^−^) and SMCs (PDGFRβ^+^tdTomato^+^). In the brain vasculature, some PDGFRβ^+^tdTomato^+^ pericytes are found at pre-capillary arterioles^6,7^. It is possible that these PDGFRβ^+^tdTomato^+^ pericytes also exist in skeletal muscle. It should be noted that these pericytes were considered as SMCs in this study due to their expression of tdTomato. As a result, their marker expression remains largely unknown and should be addressed in future research.

## Conclusion

In summary, we screened muscle pericyte-enriched and SMC-enriched genes using bulk RNAseq. By comparing pericyte-enriched genes in the brain and skeletal muscle, we further demonstrated that GSN was expressed in muscle rather than brain pericytes. This study identified GSN as a muscle pericyte-selective marker, which may be used to isolate and study muscle pericytes.

## Methods

### Animals

The SM22α-Cre mice were crossed with the Ai14 reporter mice to generate Ai14^+/−^:SM22α-Cre^+^ mice, in which SMCs were permanently labeled with tdTomato. Both the SM22α-Cre (Jax: 004746) and Ai14 reporter mice (JAX:007914) were from the Jackson Laboratory. These mice were housed in the animal facility with free access to water and food under 12 h/12 h light/dark cycle. All procedures were approved by the University of South Florida Institutional Animal Care and Use Committee (IACUC) in accordance with the National Institutes of Health Guidelines for the Care and Use of Laboratory Animals. 18 mice of both sexes at the age of 3–4 months were used in this study. Animal research is reported according to the ARRIVE guidelines.

### Muscle dissection and Preparation

Mouse skeletal muscle single cell suspension was prepared using a well-established protocol^23,41,42^. Briefly, mice were anesthetized and transcardially perfused with PBS. Hindlimb muscles were dissected and minced with sterile scissors and blades. The minced muscle fragments were digested in 0.2% (w/v) type-2 collagenase (Worthington, LS004176) at 37 °C for 2 h with rotation, followed by 3 × 15-minute incubations in 0.25% trypsin/EDTA at 37 °C. Next, tissue suspension was triturated with 1 ml pipette tips and centrifuged at 700 g for 6 min. The red blood cells (RBCs) were removed by resuspending the pellet in RBC lysis buffer and centrifugation at 700 g for 6 min. The pellet was resuspended in sorting buffer (HBSS + 2% FBS + 2mM EDTA + 1% PS) and passed through a 40-µm cell strainer to remove aggregates. The resulting single cell suspension was used for subsequent cell isolation immediately.

### Fluorescence-activated cell sorting (FACS)

Skeletal muscle single cell suspension was incubated with PDGFRβ-APC (eBioscience, 17-1402-82, 1:100), CD31-FITC (Biolegend, 103108, 1:100), and CD45-FITC (eBioscience, 11-0451-85, 1:100) for 30 min on ice. The cells were washed in ice-cold sorting buffer and resuspended in sorting buffer supplemented with DAPI. Next, the samples were filtered through a 40 μm cell strainer and run through a MoFlo Astrios EQ (Beckman Coulter) cell sorter. Single-color controls were used to perform compensation, and fluorescence-minus-one (FMO) controls were used to set the gating boundaries. Pericytes and SMCs were gated as DAPI^low^FITC^−^APC^+^tdTomato^−^ and DAPI^low^FITC^−^APC^+^tdTomato^+^ cells, respectively. Due to the relatively low density of pericytes in skeletal muscle^1^, multiple sorts were combined to generate one sample, which contained approximately 60,000 cells. Four biological replicates were used in this study.

### RNAseq and data analyses

Total RNA was extracted using the TRIzol reagent (Invitrogen, 15596018) followed by RNeasy Plus Mini Kit (Qiagen, 74136). Samples that passed RNA quality control were subjected to Ultra-Low Input RNAseq at GENWIZ. Briefly, reverse transcription and cDNA amplification were performed using the SMART-Seq v4 Ultra Low Input RNA Kit (Clontech). Library was constructed using the Illumina Nextera XT kit and sequencing (2 × 150 bp) was performed on the Illumina platform. Raw RNAseq data were provided in FASTQ format. The quality of raw RNAseq reads was assessed using the FastQC program^46^. High-quality reads were aligned to the mouse reference genome (NCBI Build 37) using Bowtie2^47^ and Tophat^48^ with default parameters and the Ensembl Gene transfer format (GTF) file. Gene expression levels were estimated in FPKM (Fragments Per Kilobase of transcript per Million mapped reads). For differential expression analysis, the LIMMA program^49^ was employed to compare gene expression levels between pericytes and SMCs. The identified DEGs were input into the Shinygo web service^50,51^ for functional or pathway enrichment analysis. The PCA plot was generated using the CummeRbund program^51^. The volcano plot was created using the Tmisc package in R programing language. The sequencing data generated from this study have been deposited in the NCBI Gene Expression Omnibus (GSE302448).

### IHC

IHC was performed as described previously^30,52^. Briefly, TA muscle sections were fixed in 4% PFA for 20 min, washed in PBS, and incubated in blocking buffer (5% normal donkey serum in PBS + 1% BSA + 0.3% Triton X-100) for 2 h at room temperature. Then, the tissues were incubated in PDGFRβ (Cell Signaling Technology, 3169 S and eBioscience, 14-1402-82, 1:200), CD13 (BD, 558744, 1:200), vimentin (Millipore, MAB1681, 1:200), SMA (Sigma, F3777, 1:1000), and GSN (R&D, MAB8170, 1:200) antibodies overnight at 4 °C. The next day, the slides were incubated in appropriate secondary antibodies for 2 h at room temperature and then mounted in Fluoromount-G with DAPI.

### Image analyses

Images were taken using the Nikon Eclipse NI-E microscope with pco.panda USB3.1 sCMOS camera and Apo Lambda 20X objective, and Agilent BioTek Cytation C10 Confocal Imaging Reader with Sony CMOS camera (I6-bit grayscale) and 40 × 1.15 NA objective. Images were acquired on NIS-Elements AR and Gene5IPRIME software. Image processing was performed using ImageJ (NIH) and Adobe Photoshop.

## Supplementary Information

Below is the link to the electronic supplementary material.

Supplementary Material 1

Supplementary Material 2

## Data Availability

Sequencing data generated from this study has been deposited in the NCBI Gene Expression Omnibus and is accessible through the accession number GSE302448. Other materials generated in this manuscript are available from the corresponding author upon reasonable request.

## Abbreviations

BBB: blood-brain barrier
DCN: decorin
DEGs: differentially expressed genes
FACS: fluorescence-activated cell sorting
FC: fold change
FMO: fluorescence minus one
GSN: gelsolin
IHC: immunohistochemistry
PCA: principal component analysis
RBCs: red blood cells
SMA: smooth muscle actin-α
SMCs: smooth muscle cells
TA: tibialis anterior
