# Supplementary material for: Screening and identification of muscle pericyte selective markers
Source: Sci Rep. 2025 Aug 7;15:28874. doi: 10.1038/s41598-025-14225-3 (PMC12332025; doi:10.1038/s41598-025-14225-3)
Supplement: Supplementary file 2 — Supplementary Material 2 [file 41598_2025_14225_MOESM2_ESM.pdf]

Screening and identification of muscle pericyte selective markers

Jingsong Ruan[1], Minkyung Kang[1,#,] Rong Wang[2], Wanling Xuan[2], Feng Cheng[2], Yao Yao[1,*]

[1]Department of Molecular Pharmacology and Physiology, Morsani College of Medicine, University of South Florida, Tampa, FL, USA

[2]Department of Pharmaceutical Sciences, Taneja College of Pharmacy, University of South Florida, Tampa, Florida, USA.

[#]Current Address: Department of Neurosurgery, Stanford University, Stanford, CA, USA

*Corresponding Author: Yao Yao, PhD, FAHA

Department of Molecular Pharmacology and Physiology

Morsani College of Medicine, University of South Florida

12901 Bruce B. Downs Blvd., MDC 8

Tampa, FL 33612, USA

Tel: 813-974-9489; Fax: 813-974-3079; Email: yao7@usf.edu

ORCID: 0000-0001-8020-9696

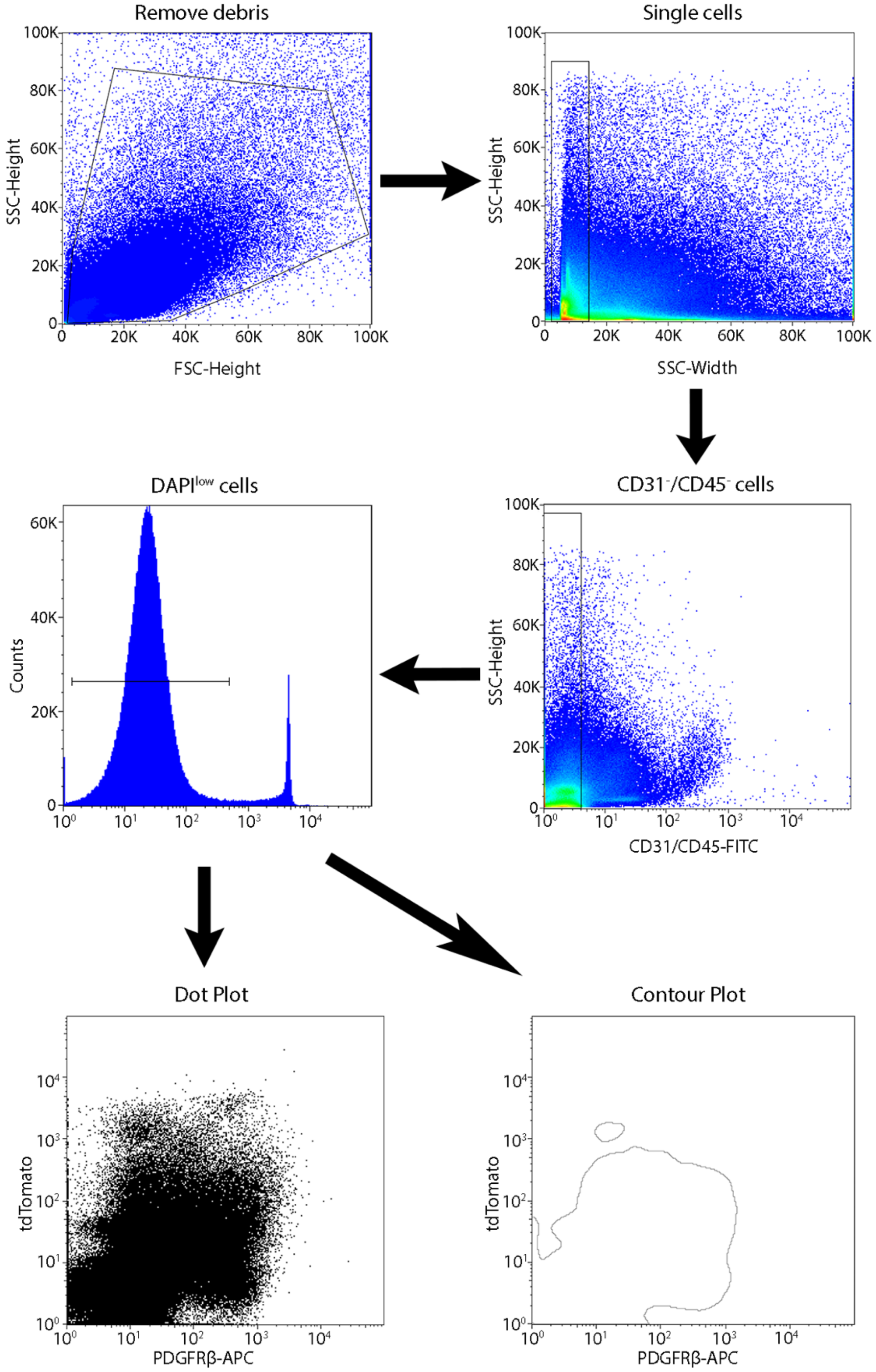

Fig. S1. Workflow and gating strategy. Flow cytometry was performed to isolate pericytes ($CD31^-CD45^-DAPI^{low}PDGFR\beta^+tdTomato^-$) and SMCs ($CD31^-CD45^-DAPI^{low}PDGFR\beta^+$ $tdTomato^+$) from skeletal muscle of $Ai14^{+/-}$;SM22α-Cre^+ mice. SMCs, smooth muscle cells.
